# The Effects of a Grape Seed Procyanidin Extract on Cytochrome P450 3A4 Activity and Inflammatory Mediators in the Lungs of Heavy Active and Former Smokers

**DOI:** 10.3390/ijms252313105

**Published:** 2024-12-06

**Authors:** Bingye Xue, Clifford Qualls, Alexander Lanthiez, Qing-Yi Lu, Jieping Yang, Ru-Po Lee, Patricia Neis, Jenny T. Mao

**Affiliations:** 1 Pulmonary, Critical Care and Sleep Section, New Mexico Veterans Administration Health Care System, University of New Mexico, Albuquerque, NM 87108, USA; bingye.xue@va.gov (B.X.); patricia.neis@va.gov (P.N.); 2 Biostatistics, Biomedical Research Institute of New Mexico, New Mexico Veterans Administration Health Care System, University of New Mexico, Albuquerque, NM 87108, USA; clifford.qualls@gmail.com; 3 Pulmonary and Critical Care Section, Veterans Administration San Diego Healthcare System, University of California San Diego, La Jolla, CA 92161, USA; lanthiez@arizona.edu; 4 Department of Pathology and Laboratory Medicine, David Geffen School of Medicine, University of California Los Angeles, Los Angeles, CA 90095, USA; qingyilu@ucla.edu; 5 UCLA Center for Human Nutrition, David Geffen School of Medicine at UCLA, Los Angeles, CA 90095, USA; jiepingyang@mednet.ucla.edu (J.Y.); rupolee@mednet.ucla.edu (R.-P.L.)

**Keywords:** leucoselect phytosome, cytochrome P450 3A4, TNF, CCL3, granzyme B, bronchoalveolar lavage, lung microenvironment, cell invasion

## Abstract

Grape seed procyanidin extract (GSE) is widely used to promote cardiovascular health and has purported anti-inflammatory properties. Chronic inflammation in the lungs caused by environmental toxins such as tobacco smoking plays a pivotal role in lung cancer development. In a modified phase I lung cancer chemoprevention study conducted in heavy active and former smokers using leucoselect phytosome (LP), a standardized grape seed procyanidin extract complexed with soy phospholipids to enhance bioavailability, three months of LP treatment favorably modulated a variety of surrogate endpoint biomarkers, including markers of cell proliferation. In this correlative study, we further analyzed the effects of LP on cytochrome P450 3A4 (CYP3A4) activities by comparing the endogenous conversions of cortisol and cortisone to 6-beta-hydroxycortisol and 6-beta-hydroxycortisone, respectively, before and after LP treatment and the anti-inflammatory effects of LP in the lung microenvironment of these participants by comparing a profile of inflammatory cytokines and chemokines in matched pre- and post-treatment bronchoalveolar lavage (BAL) fluids. LP treatment did not significantly alter CYP3A4 activity, and three months of LP treatment significantly decreased tumor necrosis factor (TNF), C-C Motif Chemokine Ligand 3 (CCL3) and granzyme B in BAL fluids. Furthermore, post-LP-treatment BAL fluids significantly reduced migration/invasion of various human lung neoplastic cells in vitro. Our findings support the anti-inflammatory effects of GSE/LP in the lung microenvironment and its potential utility for reducing cancerizing forces, as well as driving forces for other common respiratory diseases such as chronic obstructive pulmonary disease and asthma, in the lungs of heavy former and active smokers.

## 1. Introduction

The use of grapes is known to date back to Neolithic times [1]. Grapes are fruits of *Vitis vinifera* vines and are among the most consumed fruits in the world. Most commercial preparations of grape seed polyphenols, widely referred to as “grape seed extract (GSE)”, are standardized to contain ~95% procyanidins and are marketed in the USA as dietary supplements [2], mainly to promote cardiovascular health. In addition, many studies have shown anti-inflammatory, anti-arthritic, anti-allergic, and anticancer activities of GSE [3,4,5]. In preclinical studies, we demonstrated various antineoplastic effects of GSE against lung cancer [6,7,8]. We then reported the feasibility of three months of leucoselect phytosome (LP), an inexpensive standardized GSE of oligomeric procyanidins (OPCs) complexed with soy phospholipids to enhance bioavailability, for lung cancer chemoprevention in heavy former or active smokers at high risk for lung cancer. LP treatment favorably modulates a variety of surrogate endpoint biomarkers (SEBMs), including a significant reduction in bronchial Ki-67 labeling index (LI), a marker of cell proliferation on the bronchial epithelium and a key SEBM for lung cancer chemoprevention trials [9]. In addition, metabolomics analyses demonstrate that LP treatment also significantly increases fasting serum eicosapentaenoic acid (EPA) and docosahexaenoic acid (DHA), the omega-3 polyunsaturated fatty acids (n-3 PUFAs) with well-established anticancer properties [10].

Because prior preclinical research has suggested that GSE may interact with cytochrome (CYP) 450 3A4 (CYP3A4) [11], characterizing the effects of oral LP on CYP3A4 activity has important clinical implications on potential drug interactions and clinical use of LP. CYP450 enzymes are heme-containing monooxygenases responsible for the metabolism of numerous xenobiotics including therapeutic drugs, environmental chemicals, dietary constituents, and endogenous substrates such as steroids and bile acids. CYP3A4 is the most abundant isoform of the P450 family expressed in the liver and intestine [12], and it metabolizes more than 50% of clinically used drugs [13]. CYP3A4, also known as 6β-hydroxysteroid dehydrogenase, catalyzes the formation of 6β-hydroxycortisol (OHF) from cortisol (F) and 6β-hydroxycortisone (OHE) from cortisone (E) in the liver and other tissues [14]; the resulting 6β-OHF and 6β-OHE are excreted in urine. Simultaneous measurements of spot urine F and E and their respective metabolites 6β-OHF and 6β-OHE enable the determination of (6β-OHF + 6β-OHE)/(F + E) to be utilized as an endogenous biomarker for assessing the impact of a specific drug on CYP3A4 activity in vivo [15].

Chronic inflammation has been recognized to play a pivotal role in cancer development. It is well known that many cancers arise from sites of infection, chronic irritation, and inflammation [16], including lung cancers that are induced by tobacco smoking. Tobacco smoking causes acute and chronic inflammation in the lung microenvironment, orchestrated by inflammatory and immune modulatory cells that contribute to driving forces of cancerization, such as uncontrolled cell proliferation, resistance to apoptosis, and promotion of migration/invasion. Tumor cells also utilize some of the inflammatory signaling molecules from the innate immune system, such as chemokines and their receptors, for invasion, migration, and metastasis. These understanding and insights into tumor biology provide the background and rationale for developing new anti-inflammatory approaches for lung cancer chemoprevention and treatment.

Previously, we evaluated LP for its antineoplastic and chemopreventive properties against lung cancer [6,7,8,9,10]. As a part of the continuing translational research efforts on LP against lung cancer, in this correlative and biomarker-based study, we used available archived specimens with matched pre- and post-treatment spot urine samples from our phase I lung cancer chemoprevention study with LP, simultaneously measured F, E, 6β-OHF and 6β-OHE, and compared the ratios of (6β-OHF + 6β-OHE)/(F + E) to define the effects of oral LP on CYP3A4 activity. Furthermore, we evaluated the anti-inflammatory effects of oral LP treatment within the lung microenvironment of these high-risk participants by profiling and comparing levels of various cytokines and chemokines in the bronchoalveolar lavage (BAL) fluids obtained pre- and post-treatment. LP treatment did not significantly alter (6β-OHF + 6β-OHE)/(F + E), and three months of LP treatment significantly reduced TNF, CCL3, and granzyme B, correlating to a significant reduction in invasiveness of human lung cancer cell lines A549 (adenocarcinoma), H1299 (metastatic NSCLC), and H520 (squamous cell carcinoma) by co-culture cell invasion assays. Our findings further support the ongoing investigations of LP for lung cancer prevention and treatment.

## 2. Results

### 2.1. Effects of LP Treatment on Urinary (6β-OHF + 6β-OHE)/(F + E)

**Figure 1:**
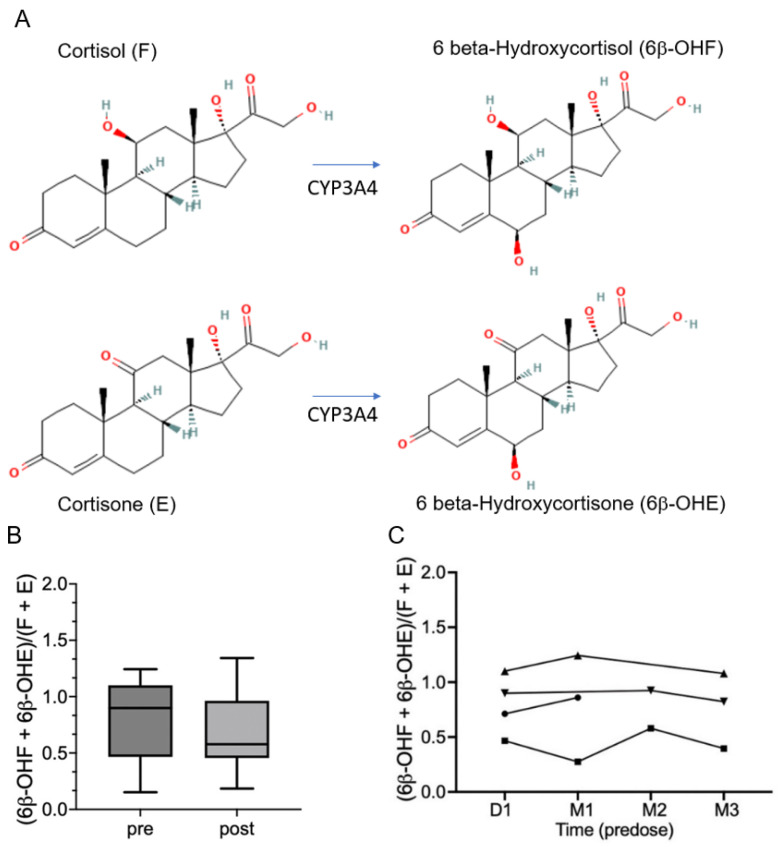
(**A**)**.** Chemical structure of F and E and their respective conversions to 6β-OHF and 6β-OHE by CYP3A4. (**B**). To determine if LP is a CYP3A4 modulator, CYP3A4 activity was evaluated by comparing urinary (6β-OHF + 6β-OHE)/(F + E) before and after treatment at various timepoints. A total of 22 samples with pre- and post-treatment comparisons were assessed. Of these, 7 sets of matched pre-dose followed by 60–120 min post-oral-LP-treatment urine samples from 5 participants were available for comparison, showing no significant acute changes post-treatment. Longitudinal comparisons of matched pre-dose urine samples at baseline (D1) vs. after 1 month (M1), 2 months (M2), or 3 months (M3) of treatment available from 4 subjects also did not show significant changes of in the ratio over time (**C**).

The effects of LP treatment on CYP3A4 activity were determined by comparing matched urinary (6β-OHF + 6β-OHE)/(F + E) before and after treatment at various timepoints. A total of 22 samples with matched pre- and post-treatment comparisons were available for measurements. Of these, seven sets of pre-dose with matched 60–120 min post-oral-LP-treatment urine samples from five subjects were available for comparison, showing no significant acute changes post-treatment (Figure 1B). Longitudinal comparisons of pre-dose urine samples at baseline, and after 1 month, 2 months, and 3 months of treatment available from four participants also did not show significant changes in urinary (6β-OHF + 6β-OHE)/(F + E) over time (Figure 1C).

### 2.2. Effects of Three Months of Oral LP Treatment on Profiles of Inflammatory Markers in the Lung Microenvironment

**Figure 2:**
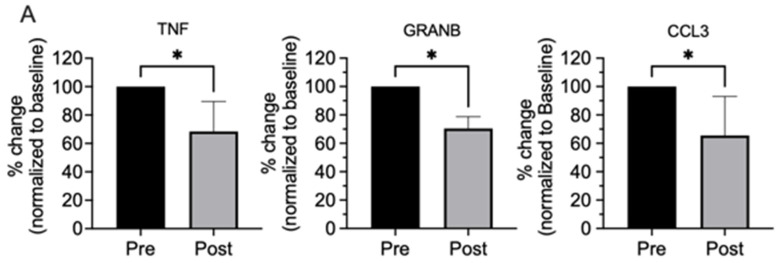

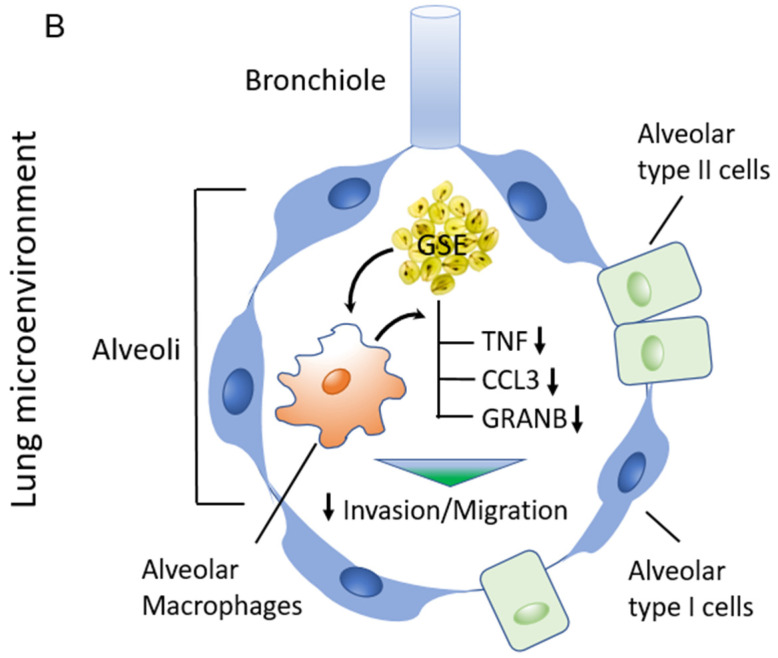
(**A**). Three months of oral LP treatment significantly decreased TNF, GRANB, and CCL3 in BAL fluids of active and former smokers available for comparative measurements. Mean; bars, SEM (n = 5, 3 heavy active and 2 heavy former smokers). *, *p* < 0.05. (**B**). Proposed mechanistic diagram of the effects of LP treatment on inflammatory mediators in the lung microenvironment of heavy former and active smokers. LP treatment decreases TNF, granzyme B, and CCL3, presumably, in part, through its action on AMs. Collectively, modulations of these inflammatory mediators create a lung microenvironment that is much less conducive to lung cancer cell invasion and migration, one of the hallmarks of the driving forces of cancerization.

To determine the effects of oral LP treatment on inflammatory protein markers within the lung microenvironment, levels in matched pre- and post-3-month-s treatment BAL samples were compared. A total of five sets of paired samples were available for evaluation. Among the 18 marker proteins (Table 1), TNF, CCL3, and granzyme B were found to be significantly altered in BAL fluids (Table 2, Figure 2A).

### 2.3. Post-Treatment BAL Fluid Samples Inhibit Cell Invasion and Migration of Human Lung Cancer Cells

**Figure 3:**
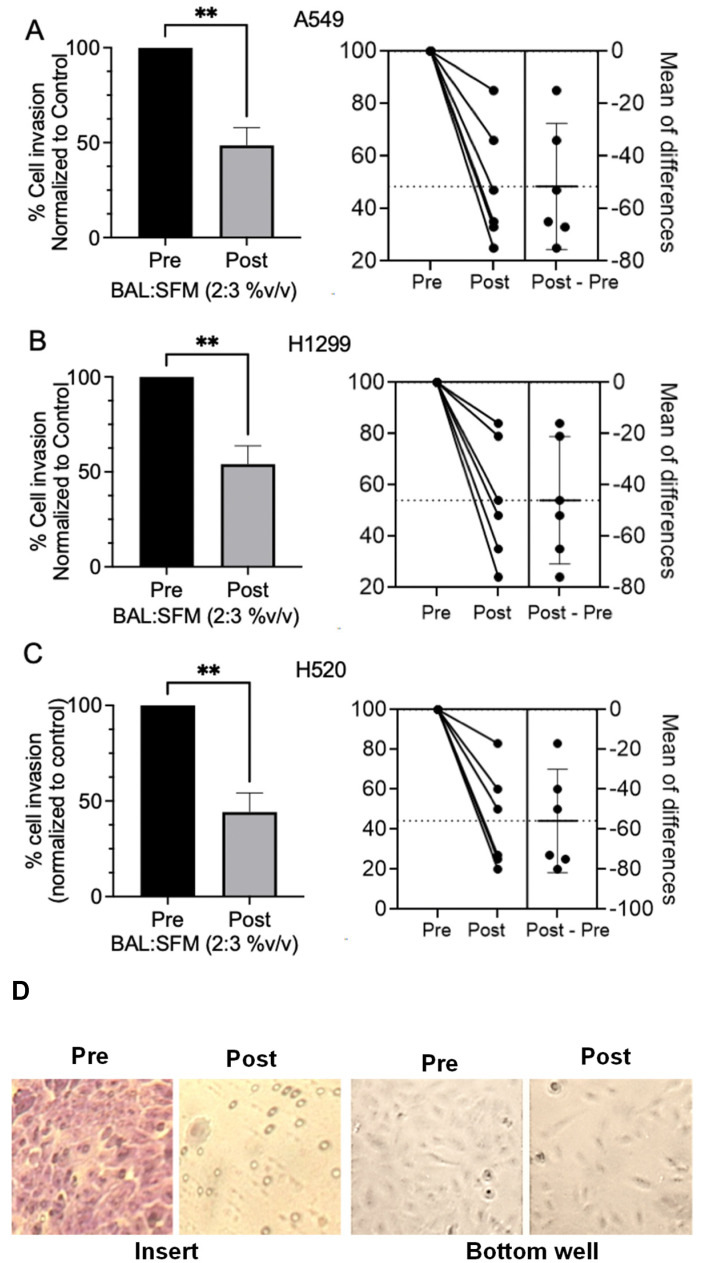
To determine the antineoplastic bioactivity of orally administered LP on cancer cell invasion/migration in the lung microenvironment, (**A**). A549, (**B**). H1299, and (**C**). H520 cells were treated with matched BAL fluids available from subjects pre- and post-3 months of LP treatment (BAL/:serum free culture medium = 2:3%*v*/*v*). Post-LP-treatment BAL fluids significantly reduced cell invasion/migration in comparison to pre-treatment BAL fluids. Percent change of invaded/migrated cells from each cell type and each participant was calculated first by normalizing matched post-treatment to baseline pre-treatment values. Mean; bars, SEM (n = 6). **, *p* < 0.01. (**D**). Representative photomicrograph of A549 cell invasion/migration assay with matched pre- and post-LP-treated BAL fluids. Left panel: Pre-LP and post-LP-treatment BAL fluid co-culture inserts following 48 h of incubation and after removal of A549 cells on the top of the insert followed by toluidine blue staining. The insert conditioned with post-LP BAL fluids showed mostly 8 μM pores as the non-invaded cells had been mechanically removed. Right panel: Migrated cells at the bottom of the transwell after 96 h incubation. Significantly fewer cells were seen when conditioned with matched post-LP BAL fluid, consistent with less migration/invasion.

To further demonstrate functional, anti-neoplastic effects of oral LP treatment in the lung microenvironment, cell invasion/migration assays were performed on A549, H1299, H520, and DMS114 cells using a transwell co-culture system with matched pre- and post-LP-treatment BAL fluids. Post-LP-treatment BAL fluids significantly inhibited cell invasion/migration of NSCLC cell A549, H1299, and H520 NSCLC cells in comparison with matched pre-treatment BAL fluids (Figure 3). Post-LP-treatment BAL fluids, however, did not significantly decrease cell invasion/migration of small cell lung cancer DMS114 cells. 

## 3. Discussion

In this study, we demonstrate that acute and chronic oral administration of LP does not significantly impact CYP3A4 activity through simultaneous measurements of urinary (6β-OHF + 6β-OHE)/(F + E) before and after treatment at various timepoints. This finding has important, practical implications for the clinical applicability of LP. For example, LP is mainly used as a health food supplement to promote cardiovascular health. Patients with these diseases are often on multiple medications, many of which are CYP3A4 substrates that may have a narrow therapeutic index, and some may have active metabolites generated by CYP3A4. Inhibition or activation of CYP3A4 can increase or decrease the bioavailability of drugs that are CYP3A4 substrates, thereby increasing toxicity or diminishing the effectiveness of these drugs, respectively. Our findings suggest LP does not significantly impact overall CYP3A4 activities and is therefore not contraindicated with concomitant administration of oral medications known to be CYP3A4 substrates. To our knowledge, this is the first report of the impact of GSE/LP on CYP3A4 activities in humans.

BAL is a procedure commonly employed to obtain samples from the lungs. BAL fluid and cells can be readily obtained during bronchoscopy, and they represent a relatively noninvasive means of sampling the cellular/molecular components of the lung microenvironment. Human BAL cells usually comprise ~90% alveolar macrophages (AMs). AMs are the predominant cell type within the alveoli and serve as the first line of host defense against inhaled organisms and soluble and particulate molecules, including those present in tobacco smoke. AMs play a complex immunoregulatory role in the lung microenvironment. These immunoeffector cells have been shown to produce a wide variety of pro- and anti-inflammatory agents [17]. Through the elaboration of these biologic response modifiers, AMs play a central role in mediating immune surveillance and antitumor immunity in the lung microenvironment. Many of the functions of alveolar macrophages have been shown to be dysregulated following exposure to cigarette smoke, thereby contributing to smoking-related diseases [18]. As such, BAL fluid offers an important surrogate model system for studies of the lung microenvironment in response to antineoplastic agents for lung cancer prevention and treatment [19,20]. To this end, we profiled common inflammatory mediators including cytokines and chemokines (Table 2) in BAL fluids from our participants, which are known to be involved in inflammation and tumor biology. We demonstrate that 3 months of once-daily oral LP treatment significantly decreases pro-inflammatory TNF, CCL3, and granzyme B in BAL fluids. Post-treatment BAL fluid samples also significantly inhibit cell invasiveness of a variety of human lung cancer cell lines in vitro in comparison to matched pre-treatment BAL samples.

TNF (also referred to as TNF-alpha) is a potent, pleiotropic pro-inflammatory cytokine that plays a pivotal role in initiating a cascade of activation of other cytokines and growth factors in inflammatory responses. Secreted by a variety of immune effector cells including macrophages, TNF affects almost any type of cell, including macrophages themselves [21]. TNF promotes the inflammatory activity of macrophages but also controls macrophage survival and death. Initially identified in the late 1970s as a cytokine produced by immune cells having the capacity to suppress tumor cell proliferation and induce tumor regression [22], TNF has also been reported to function as an endogenous tumor promoter, capable of stimulating cancer cell growth, proliferation, invasion, and metastasis, as well as tumor angiogenesis [23]. Both exogenous and macrophage-produced TNF accelerate the epithelial–mesenchymal transition (EMT), a mechanism that characterizes the progression of carcinoma and has been linked to the acquisition of an invasive phenotype [24,25,26]. Furthermore, TNF has been reported to induce chemokines, including CCL-3 [27].

CCL3, also known as macrophage inflammatory protein-1α (MIP-1α), is a member of the CC chemokine family. The involvement of CCL3 has been reported in the progression of various malignancies. For example, the axis of CCL3 and one of its receptors, CCR5, has been associated with lung metastasis in murine renal cell carcinoma [28]. In addition, CCL3 derived from both tumor-associated macrophages (TAMs) and cancer cells contributes to the progression and poor prognosis of esophageal squamous cell carcinoma by promoting cell migration and invasion via the binding of CCR5; the CCL3-CCR5 axis induces migration of esophageal squamous cell carcinoma via the activation of Akt and ERK signaling pathways [29]. Moreover, CCL3 plays a vital role in promoting EMT via the PI3K-protein kinase B (Akt)-mTOR signaling pathway in breast cancer cells when co-cultured with myeloid-derived suppressor cells [30].

Granzyme B is a serine protease traditionally known to be produced by cytotoxic lymphocytes and functions as a major mediator of the cytotoxic immune response by inducing target cell death when internalized in the presence of perforin. More recently, multiple extracellular functions of granzyme B have been identified, including its capability of cleaving extracellular matrix (ECM) components, cytokines, cell receptors, and clotting proteins. These findings support the potential multifunctional, pro-inflammatory nature of granzyme B in contributing to the pathogenesis of various inflammatory diseases and cancer [31]. Granzyme B has been shown to be produced by other cell types and implicated in extracellular matrix (ECM) remodeling and degradation of ECM proteins. For example, macrophages have been shown to express granzyme B in the lesion areas of atherosclerosis and rheumatoid arthritis [32]. Granzyme B has been reported to be an up-modulator of EMT and invasion in colorectal cancer cells, and DHA inhibits urothelial, pancreatic, and colorectal carcinoma cell invasion and their granzyme B expression [33]. Based on our recently reported findings that LP increases serum DHA in our participants [10], it is plausible that DHA may have contributed to the inhibition of granzyme B in the lung microenvironment as well. Granzyme B has also been reported to promote proliferation, migration, and EMT processes in gastric cancer [34].

Collectively, our findings suggest that treatment with LP decreases chronic inflammation in the lung microenvironment, as evidenced by a reduction in predominantly AM-derived TNF, which in turn decreases CCL3 in the BAL fluid. LP treatment also likely decreases granzyme B secretions by AMs. Alternatively, LP may modulate each of these biologic response modifiers through direct action on AMs (Figure 2B), and/or through other immunoeffector cells that typically constitute < 10% of the BAL cell population. Ultimately, reductions in these mediators translate into a lung microenvironment that is less conducive to invasiveness, as evidenced by a reduction in invasion/migration of a variety of NSCLC cell lines co-cultured with BAL fluids. Interestingly, LP-treated BAL fluids do not significantly reduce the invasiveness of DMS114, which may be due to the more invasive nature of SCLC.

In addition, we identify trends toward significant decreases of interleukin (IL)-6 and C-X-C motif chemokine ligand 10 (CXCL10), as well as a trend toward an increase in IL-12p70 in BAL fluids by LP treatment. Whereas the limited sample sizes are insufficient to demonstrate statistically significant modulations of these inflammatory mediators that are also known to be involved in tumor biology [35,36,37,38], these findings are nonetheless intriguing and remain to be elucidated in future clinical trials with LP.

In summary, our findings support the notion that oral administration of GSE/LP does not significantly modulate CYP3A4 activity and illustrate the potential anti-inflammatory and chemopreventive effects of GSE/LP in the lung microenvironment, through favorable modulations of key mediators of inflammatory response in the lungs. These findings, along with the significant reduction in Ki-67 LI in the proximal bronchi, and favorable modulations of a variety of other eicosanoids, as well as a reduction in lung cancer cell proliferation by post-LP-treatment BAL fluids from our previous reports [10], further support the potential of oral LP in dampening the driving forces of cancerization in the lungs. Our findings also suggest that LP may benefit other common respiratory diseases such as chronic obstructive pulmonary disease (COPD) and asthma via anti-inflammatory effects by reductions in these pro-inflammatory mediators, as TNF, CCL3, and granzyme B have all been implicated in playing important roles in the pathophysiology of asthma and COPD [39,40,41,42,43,44,45,46,47].

We acknowledge that our study has limitations. We are unable to address the potential impact of GSE/LP treatment on IFN-γ, IL-2, -4, 5, -17A, and CCL7 levels in the lung microenvironment as they are below the detection limits of ELLA, likely due to the dilutional effect of BAL. Furthermore, our findings, while intriguing, are based on limited sample sizes from a single-arm, modified phase 1 study and must be validated in larger, double-blind, randomized, placebo-controlled clinical trials to confirm the utility of LP as an antineoplastic and chemopreventive agent against lung cancer.

## 4. Materials and Methods

### 4.1. Leucoselect Phytosome Clinical Study Design

A modified phase I lung cancer chemoprevention study of 3 months of oral LP, comprising standardized OPCs complexed with soy phospholipid or lecithin (1:2.6 *w*/*w*; Indena, Milan, Italy, supplied via Thorne Research), was conducted in high-risk heavy active or ex-smokers 21 years of age or older with a smoking history of at least 30 pack-years (pky) as previously described (Table 3 and Table 4) [9]. Written informed consent was obtained in accordance with the New Mexico VA Health Care System (NMVAHCS) Institutional Review Board, following the guidelines of the Declaration of Helsinki, Belmont Report, and U.S. Common Rules. Qualified participants were treated with 1 capsule (cap), 450 mg/cap once a day, escalating weekly to 4 caps once a day for the rest of the treatment duration as tolerated. Spot urine samples were collected at baseline, before and 60–120 min after oral LP on day 1, and at the beginning of each month’s follow-up visit and stored at −80 °C. Fluorescence bronchoscopy with BAL and bronchial biopsies were performed at baseline and at the end of 3 months of treatment as previously described [19,20]. Briefly, BAL was performed by wedging the bronchoscope into the subsegment of the right middle lobe, followed by instillations of four 60 mL aliquots of room temperature saline serially and recovered by manual syringe suction. Recovered fluid was passed through a Falcon 100-micron sterile nylon filter (Corning, Glendale, AZ, USA)) to remove mucus and particulates, pooled, and centrifuged at 300× *g* for 8 min at 4 °C. The BAL fluid was then harvested, aliquoted, and stored at −80 °C until analyzed.

### 4.2. Measurement of Urinary 6β-Hydroxycortisol (OHF), Cortisol (F), 6β-Hydroxycortisone (OHE), and Cortisone (E)

6β-OHF, F, 6β-OHE, and E were purchased from Sigma Chemical Co. (St. Louis, MO, USA) and 6b-OHE from Santa Cruz Biotechnology Inc. (Dallas, TX, USA). They were of at least 98% purity. Acetonitrile and methanol used were of liquid chromatography (LC) grade and purchased from Fisher Scientific. Preparations for standards F, E, 6β-OHF, and 6β-OHE were reconstituted as a 1 mg/mL solution in methanol. All stock solutions were stored at –20 °C.

Five hundred µL of urine was spiked with 10 µL of 1 µg/mL of internal standards 6b-OHF d4, F d4, and E d8 (CDN isotopes) to achieve a final concentration of 20 ng/mL. Methyl tert-butyl ether (MTBE) (500 µL) was added, and the mixture was vortexed for 10 min. After centrifugation at 14,000 rpm for 10 min, the upper layer was collected. The MTBE extraction was repeated once more, and all MTBE extracts were combined and dried using a speed vacuum. The residue was then dissolved in 100 µL of 50% methanol solution and injected into the LC–mass spectrometry/mass spectrometry (LC-MS/MS) system. The analysis was performed on a Thermo Finnigan triple quadrupole TSQ Quantum MS/MS system (San Jose, CA, USA), equipped with an Agilent 1100 series LC system. Chromatographic separation was achieved on an Agilent Zorbax SB-C18 2.1 × 150 mm column (Santa Clara, CA, USA). Eluent A consisted of 0.1% formic acid in acetonitrile, and Eluent B consisted of 0.1% formic acid in water. Gradient elution was performed starting at 90% B with a flow rate of 0.25 mL/min, linearly decreasing to 30% B over 30 min. Due to the stable adduct formation with formic acid in ESI (-) mode (M + HCOO)- the MS and MS/MS parameters were optimized for F (407/331), E (405/329), 6β-OHF (423/347), 6β-OHE (421/345), F d4 (411/335), E d8 (413/335), and 6β-OHF d4 (427/351) [48].

### 4.3. ELLA Simple Plex Assays

The ELLA Simple Plex System using customized microfluidic Simple Plex cartridges with preloaded, specific capture antibodies to a panel of 18 markers of interest (Table 2) was used to profile the concentrations of these marker proteins in BAL fluids per the manufacturer’s instruction. The system uses fluorescence detection, and each microfluid channel has three Glass Nano Reactors coated with a specific capture antibody yielding triplicate results for each sample. Calibration curves are preloaded on the cartridges, and the mean concentration for each specific maker is automatically generated by the system (R&D Systems, biotechne, Minneapolis, MN, USA). Concentrations of markers in BAL samples were further normalized to concentrations of albumin in BAL fluids to control for BAL inter-procedural variations.

### 4.4. Cell Cultures

As models for evaluating the antineoplastic bioactivity of oral LP against lung cancer, the human non-small-cell lung cancer (NSCLC) cell lines A549, H1299, and H520, and the human small cell lung cancer cell (SCLC) line DMS114 (purchased from ATCC; Manassas, VA, USA), were co-cultured in vitro with matched, pre- and post-3-month-treatment BAL fluids from study participants. Experiments involving A549 were initiated within 6 months of purchase. ATCC uses Short Tandem Repeat profiling for cell line authentication. A549, H1299, H520, and DMS114 cells were not further authenticated. Cells were last tested for mycoplasma within 6 months of the experiments. Cells were maintained as monolayers in an atmosphere of 5% CO_2_ in air at 37 °C in 25 cm^2^ tissue culture flasks containing cell-line-specific culture medium as previously described [6,7,8]. Only cells within passages 3–6 at 70–80% confluence were used.

### 4.5. Cell Invasion Assays

To quantify cell invasiveness in conditioned cells, aliquots (200 μL) of 1.5 × 10^5^ cells/mL of A549, H1299, H520, or DMS114 cells in a respective cell-line-specific serum-free medium (SFM) were plated on 24 well co-culture inserts containing 8 μM pores that were precoated with Matrigel (Corning Biocoat, Fisher Scientific, Pittsburgh, PA, USA). The inserts were then combined with the 24-well culture plate containing 300 mL of a mixture of matched pre- or post-treatment BAL fluid and SFM (2:3%*v*/*v*). After 48–72 h incubation, cells remaining on the top layer of the culture insert were removed by cotton swabs, and cells that had migrated/invaded through the matrigel were counted in at least 5 randomly selected fields. Following 96–108 h incubation, cells on the bottom wells were also counted. Cells on the insert were fixed with 100% methanol and stained with 1% toluidine blue.

### 4.6. Statistical Analysis

The effects of LP treatment on CYP3A4 activity were determined by comparing endogenous urinary (6β-OHF + 6β-OHE)/(F + E) before and after treatment at various timepoints using paired *t* tests. The effects of LP treatment on protein markers of inflammation were determined by comparing pre-treatment baseline values with those obtained at the end of 3 months of treatment. Percent changes, defined as the mean average of post-treatment divided by the mean average of matched pre-treatment values, were calculated for each marker. The functional effects of LP treatment on human lung cancer cell invasions were determined by comparing matched pre- and post-3-month-treatment values from each of the 6 participants who completed 3 months of treatment; the percent change of each biomarker from each participant was calculated first by normalizing matched post-treatment to baseline pre-treatment values, followed by paired *t* tests. Data were expressed as the mean ± SEM in all circumstances where mean values were compared. Differences were considered significant when *p* < 0.05.

## Figures and Tables

**Table 1:** A panel of inflammatory markers profiled in matched pre- and post-LP-treatment BAL fluids.

Cytokines			Chemokines	Serine Protease
IFN-γ *	IL-1b	IL-7	CCL2	Granzyme B
TNF	IL-2 *	IL-12p70	CCL3	
	IL-4 *	IL-15	CCL4	
	IL-5 *	IL-17A *	CCL7 *	
	IL-6	IL-18	CXCL10	

* Below detection range in BAL fluids.

**Table 2:** Three months of LP treatment significantly modulated three inflammatory markers, TNF, granzyme B (GRANB), and CCL3, in the lung microenvironment. All concentrations are in pg/mL.

BAL Marker	Pre-LP Range	Post-LP Range	Pre-LP Mean	Post-LP Mean	Mean % Change	*p* Value
TNF	0.191–0.848	0.13–0.459	0.451 ± 0.13	0.27 ± 0.055	40% decrease	0.0104
GRANB	2.53–6.09	2.0–3.04	3.756 ± 0.646	2.464 ± 0.166	35% decrease	0.0242
CCL3	0.893–6.18	0.741–3.51	3.043 ± 1.006	1.9 ± 0.455	38% decrease	0.0488

**Table 3:** Study entry criteria.

Inclusion
Age over 21.
Smoking history > 30 pack years.
Exclusion
Inability to provide informed consent (e.g., cognitive impairment, severe psychiatric disorders).
Hypersensitivity to grapes and related products.
Liver dysfunction (abnormal liver function tests).
Renal dysfunction (abnormal serum creatinine).
End-stage respiratory disease (FEV1 < 0.8 L, resting or exertional hypoxemia, to select patients with adequate reserve to undergo bronchoscopy and complete the study).
Unstable angina.
Malignancy within 5 years, excluding non-melanoma type skin cancer or stage I NSCLC after curative resection without evidence of recurrence.
Pregnancy.
Systemic corticoid steroid therapy.
Coagulopathy.
Concurrent use of grapes or grape-related products.
Unwilling to refrain from drinking more than 1 glass of wine a day.
Patients with concurrent medical conditions that may interfere with completion of tests, therapy, or the follow-up schedule.
Inability to provide informed consent (e.g., cognitive impairment, severe psychiatric disorders).

**Table 4:** Baseline subject characteristics.

Variables	Mean (Range)	n
Age (y)	58.6 (46–68)	
Smoking history (pack years) *	40.4 (30–55)	
Gender (M/F)		5/3
Ethnicity (Caucasian)		8
COPD (n)		2
Family history of lung cancer (n)		2

* Three subjects were active smokers.

## Data Availability

The raw data supporting the conclusions of this article will be made available by the authors on request.

## Abbreviations

LP	Leucoselect phytosome
GSE	Grape seed procyanidin extract
CYP3A4 BAL	Cytochrome P450 3A4 enzyme Bronchoalveolar lavage
TNF	Tumor necrosis factor
CCL3	C-C Motif Chemokine Ligand 3
OPCs	Oligomeric procyanidins
SEBMs	Surrogate endpoint biomarkers
Ki-67 LI	Ki-67 labeling index
EPA	Eicosapentaenoic acid
DHA	Docosahexaenoic acid
n-3 PUFAs	Omega-3 polyunsaturated fatty acids
COPD	Chronic obstructive pulmonary disease
NSCLC	Non-small-cell lung cancer
6β-OHF	6β-hydroxycortisol
6β-OHE	6β-hydroxycortisone
F	Cortisol
E	Cortisone
NMVAHCS	New Mexico VA Health Care System
SCLC	Small cell lung cancer
SFM	Serum-free medium
LC	Liquid chromatography
MS	Mass spectrometry
MTBE	Methyl tert-butyl ether
AMs	Alveolar macrophages
EMT	Epithelial–mesenchymal transition
TAMs	Tumor-associated macrophages
ECM	Extracellular matrix
IL	Interleukin
CXCL10	Chemokine ligand 10
